# Motion Compensation for Radar Terrain Imaging Based on INS/GPS System

**DOI:** 10.3390/s19183895

**Published:** 2019-09-10

**Authors:** Michal Labowski, Piotr Kaniewski, Piotr Serafin

**Affiliations:** Faculty of Electronics, Institute of Radioelectronics, Military University of Technology, ul. gen. S. Kaliskiego 2, 00-908 Warszawa, Poland

**Keywords:** INS, GPS, SAR, Kalman filter

## Abstract

In order to obtain good quality radar terrain images using an aerial-based synthetic aperture radar, a motion compensation procedure must be applied. This procedure can use a precise navigation system in order to determine the aircraft’s position and velocity. A major challenge is to design a motion compensation procedure that can operate in real time, which is crucial to ensure convenient data for a human analyst. The article discusses a possibility of Inertial Measurement System (INS)/Global Positioning System (GPS) navigation system usage in such a radar imaging system. A Kalman filter algorithm designed for this system is described herein, and its modifications introduced by the authors allow the use of navigational data not aligned in time and captured with different frequencies. The presented navigation system was tested using measured data. Radar images obtained with the INS/GPS-based motion compensation system were compared to the INS-only results and images obtained without navigation corrections. The evaluation results presented in the paper show that the INS/GPS system allows for better reduction of geometric distortions in images compared to the INS-based approach, which makes it more suitable for typical applications.

## 1. Introduction

Obtaining high-quality terrain images using aerial synthetic aperture radar (SAR) requires several conditions to be met. Firstly, the aircraft that carries the radar should move along an assumed nominal trajectory of flight, which in most cases is rectilinear. Secondly, the vehicle’s velocity must be constant during imaging. Failure to meet these requirements significantly increases the computational complexity or even makes imaging not possible. Miniature unmanned aerial vehicles (UAV) have a small mass and a limited maximum speed; therefore, meeting these requirements is extremely difficult in practical implementations. As a result, during radar data processing, it is necessary to apply algorithms aimed to compensate the radar position and the velocity deviations, referred as the motion compensation (MOCO).

The compensation can be achieved in two different ways. The first group of methods uses additional navigational equipment to determine UAV position and velocity deviations and to compute phase corrections for the received echo signals [1,2,3]. These corrections are then introduced to the echo signals in an azimuth compression procedure [4]. The second group of methods focuses on the analysis of the echo signals received by the radar sensor. The methods acting this way are referred to as the autofocus [5,6,7,8,9]. They allow the user to achieve better results in terms of image focus but are more computationally demanding than navigational methods, which makes them difficult to use in real-time imaging systems.

The basic navigation system used to improve the quality of radar terrain images is the Inertial Measurement System (INS) [10,11,12]. Echo signals corrections determined using INS allow one to reduce geometric distortions of images; however, they can be used only for short measuring sessions [10], which is not convenient in real-world applications, where it is important to have smooth and undistorted images during long-term observation missions. The allowed duration of a session varies from dozen to several tens of seconds depending on the class of the inertial unit used, and it is related to the process of INS error accumulation.

The mentioned property of the INS prompted the authors to study and develop an integrated navigation system combining INS and Global Positioning System (GPS) to estimate the UAV position and velocity deviations. In the proposed system, the INS and the GPS data integration was implemented using a Kalman filter [13,14,15,16]. The algorithm proposed by the authors was modified in comparison to the solutions used thus far in MOCO procedures [1,2,3]. In these systems, it is commonly assumed that INS and GPS receivers provide measurements with the same frequency and their solutions are available at the same moment of time. In general, however, these frequencies are different, which affects the measurement update phase of the filter. Moreover, the lack of INS and GPS synchronization makes it impossible to properly compute the measurement vector directly from INS and GPS outputs. Proposals for solving these issues are presented in the following sections of this article.

The layout of the remainder of this paper is as follows. A mathematical description of the INS/GPS system is given in Section 2. The problem of the lack of INS and GPS synchronization and its proposed solution is also described here. The proposed algorithm of a modified Kalman filter is described in detail in Section 3, and a procedure of tuning its parameters is discussed in Section 4. Chosen results of testing the designed INS/GPS system using measured data are presented in Section 5 and Section 6. The obtained results are discussed in Section 7.

## 2. System Model

When developing a Kalman filter, it is necessary to formulate a system model consisting of a dynamics model and an observation model. The dynamics model describes how the system state vector x(k) changes in time [13]:

(1) x(k)=Φ(k,k−1)x(k−1)+w(k−1)

where k is an index of discrete time, Φ(k,k−1) is a state transition matrix from k−1 to k moment, and w(k) is the process noise vector. The observation model shows a relationship between the state vector and the measurement vector z(k) [15]:

(2) z(k)=H(k)x(k)+v(k)

where v(k) is the measurement noise vector and H(k) is the observation matrix. In the proposed system, the state vector consists of INS errors:

(3) $$x={\left[\begin{array}{ccccccccc} \delta n & \delta v_{is1,N}^{n} & \phi_{E} & \delta e & \delta v_{is1,E}^{n} & \phi_{N} & \delta d & \delta v_{is1,D}^{n} & \phi_{D} \end{array}\right]}^{T}$$

where $\left[\begin{array}{ccc} \delta n & \delta e & \delta d \end{array}\right]$ is the position errors vector in the *North-East-Down* (NED) reference frame, also named *n-frame* [11], $\left[\begin{array}{ccc} \delta v_{is1,N}^{n} & \delta v_{is1,E}^{n} & \delta v_{is1,D}^{n} \end{array}\right]$ is the velocity errors vector in the sensor’s frame (*s-frame*) with reference to the inertial frame (*i-frame*) expressed in the *n-frame*, and $\left[\begin{array}{ccc} \phi_{N} & \phi_{E} & \phi_{D} \end{array}\right]$ is the orientation errors vector in the *n-frame*.

The dynamics model was determined using equations described in [15,17]. Finally, the transition matrix Φ(k,k−1) has the following form:

(4) $$\Phi =[\begin{array}{ccccccccc} 1 & T_{p} & -\frac{f_{is1,D}^{n}T_{p}^{2}}{2} & 0 & 0 & 0 & 0 & 0 & \Phi_{19}\\ 0 & \Phi_{22} & -f_{is1,D}^{n}T_{p} & 0 & 0 & 0 & 0 & 0 & \Phi_{29}\\ 0 & -\frac{T_{p}}{R} & \Phi_{33} & 0 & 0 & 0 & 0 & 0 & \Phi_{39}\\ 0 & 0 & 0 & 1 & T_{p} & \frac{f_{is1,D}^{n}T_{p}^{2}}{2} & 0 & 0 & \Phi_{49}\\ 0 & 0 & 0 & 0 & \Phi_{55} & f_{is1,D}^{n}T_{p} & 0 & 0 & \Phi_{59}\\ 0 & 0 & 0 & 0 & \frac{T_{p}}{R} & \Phi_{66} & 0 & 0 & \Phi_{69}\\ 0 & 0 & 0 & 0 & 0 & 0 & 1+\frac{g_{b,D}^{n}T_{p}^{2}}{R} & 1 & 0\\ 0 & 0 & 0 & 0 & 0 & 0 & \frac{2g_{b,D}^{n}T_{p}}{R} & 1+\frac{g_{b,D}^{n}T_{p}^{2}}{R} & 0\\ 0 & 0 & 0 & 0 & 0 & 0 & 0 & 0 & 1 \end{array}]$$

where:

(5) $$\Phi_{19}=\frac{f_{is1,E}^{n}T_{p}^{2}}{2}$$

(6) $$\Phi_{22}=1+\frac{f_{is1,D}^{n}T_{p}^{2}}{2R}$$

(7) $$\Phi_{29}=f_{is1,E}^{n}T_{p}-\frac{f_{is1,D}^{n}{\Delta \Theta }_{is1,N}^{n}T_{p}}{2}$$

(8) $$\Phi_{33}=1+\frac{f_{is1,D}^{n}T_{p}^{2}}{2R}$$

(9) $$\Phi_{39}=-\frac{f_{is1,E}^{n}T_{p}^{2}}{2R}+{\Delta \Theta }_{is1,N}^{n}$$

(10) $$\Phi_{49}=-\frac{f_{is1,N}^{n}T_{p}^{2}}{2}$$

(11) $$\Phi_{55}=1+\frac{f_{is1,D}^{n}T_{p}^{2}}{2R}$$

(12) $$\Phi_{59}=-f_{is1,N}^{n}T_{p}-\frac{f_{is1,D}^{n}{\Delta \Theta }_{is1,E}^{n}T_{p}}{2}$$

(13) $$\Phi_{66}=1+\frac{f_{is1,D}^{n}T_{p}^{2}}{2R}$$

(14) $$\Phi_{69}=-\frac{f_{is1,N}^{n}T_{p}^{2}}{2R}-{\Delta \Theta }_{is1,E}^{n}$$

and where $f_{is1}^{n}=\left[\begin{array}{ccc} f_{is1,N}^{n} & f_{is1,E}^{n} & f_{is1,D}^{n} \end{array}\right]$ is the specific force vector of the *s-frame* in relation to the *i-frame*, expressed in the *n-frame*, $\Delta \Theta_{is1}^{n}$ is the delta angle vector, R is the WGS-84 ellipsoid mean radius [9], $T_{p}$ is the period of acquiring new INS data (which equals Kalman filter prediction phase period), and $g_{b,D}^{n}$ is a vertical component of the Earth’s gravity vector.

The process noise covariance matrix Q(k−1), which must be known for implementing a Kalman filter, was determined using modified equations from [15,17]:

(15) $$Q=[\begin{array}{ccccccccc} Q_{11} & Q_{12} & Q_{13} & Q_{14} & Q_{15} & Q_{16} & 0 & 0 & Q_{19}\\ Q_{12} & Q_{22} & Q_{23} & Q_{24} & Q_{25} & Q_{26} & 0 & 0 & Q_{29}\\ Q_{13} & Q_{23} & Q_{33} & Q_{34} & Q_{35} & Q_{36} & 0 & 0 & Q_{39}\\ Q_{14} & Q_{24} & Q_{34} & Q_{44} & Q_{45} & Q_{46} & 0 & 0 & Q_{49}\\ Q_{15} & Q_{25} & Q_{35} & Q_{45} & Q_{55} & Q_{56} & 0 & 0 & Q_{59}\\ Q_{16} & Q_{26} & Q_{36} & Q_{46} & Q_{56} & Q_{66} & 0 & 0 & Q_{69}\\ 0 & 0 & 0 & 0 & 0 & 0 & \frac{S_{vD}T_{p}^{3}}{3} & \frac{S_{vD}T_{p}^{2}}{2} & 0\\ 0 & 0 & 0 & 0 & 0 & 0 & \frac{S_{vD}T_{p}^{2}}{2} & S_{vD}T_{p} & 0\\ Q_{19} & Q_{29} & Q_{39} & Q_{49} & Q_{59} & Q_{69} & 0 & 0 & S_{\phi D}T_{p} \end{array}]$$

where:

(16) $$Q_{11}=\frac{S_{vN}T_{p}^{3}}{3}+\frac{{\left(f_{is1,D}^{n}\right)}^{2}S_{\phi E}T_{p}^{5}}{20}+\frac{{\left(f_{is1,E}^{n}\right)}^{2}S_{\phi D}T_{p}^{5}}{20}$$

(17) $$Q_{12}=\frac{S_{vN}T_{p}^{2}}{2}+\frac{{\left(f_{is1,D}^{n}\right)}^{2}S_{\phi E}T_{p}^{4}}{8}+\frac{{\left(f_{is1,E}^{n}\right)}^{2}S_{\phi D}T_{p}^{4}}{8}$$

(18) $$Q_{13}=-\frac{S_{vN}T_{p}^{3}}{3R}-\frac{f_{is1,D}^{n}S_{\phi E}T_{p}^{3}}{6}+\frac{f_{is1,E}^{n}{\Delta \Theta }_{is1,N}^{n}S_{\phi D}T_{p}^{3}}{8}$$

(19) $$Q_{14}=-\frac{f_{is1,E}^{n}f_{is1,N}^{n}S_{\phi D}T_{p}^{5}}{20}$$

(20) $$Q_{15}=-\frac{f_{is1,E}^{n}f_{is1,N}^{n}S_{\phi D}T_{p}^{4}}{8}$$

(21) $$Q_{16}=-\frac{f_{is1,E}^{n}{\Delta \Theta }_{is1,E}^{n}S_{\phi D}T_{p}^{3}}{8}-\frac{f_{is1,E}^{n}f_{is1,N}^{n}S_{\phi D}T_{p}^{5}}{20R}$$

(22) $$Q_{19}=-\frac{f_{is1,E}^{n}S_{\phi D}T_{p}^{3}}{6}$$

(23) $$Q_{22}=S_{vN}T_{p}+\frac{{\left(f_{is1,D}^{n}\right)}^{2}S_{\phi E}T_{p}^{3}}{3}+\frac{{\left(f_{is1,E}^{n}\right)}^{2}S_{\phi D}T_{p}^{3}}{3}$$

(24) $$Q_{23}=-\frac{S_{vN}T_{p}^{2}}{2R}-\frac{f_{is1,D}^{n}S_{\phi E}T_{p}^{2}}{2}+\frac{f_{is1,E}^{n}{\Delta \Theta }_{is1,N}^{n}S_{\phi D}T_{p}^{2}}{3}$$

(25) $$Q_{24}=-\frac{f_{is1,E}^{n}f_{is1,N}^{n}S_{\phi D}T_{p}^{4}}{8}$$

(26) $$Q_{25}=-\frac{f_{is1,E}^{n}f_{is1,N}^{n}S_{\phi D}T_{p}^{3}}{3}$$

(27) $$Q_{26}=-\frac{f_{is1,E}^{n}{\Delta \Theta }_{is1,E}^{n}S_{\phi D}T_{p}^{2}}{3}-\frac{f_{is1,E}^{n}f_{is1,N}^{n}S_{\phi D}T_{p}^{4}}{8R}$$

(28) $$Q_{29}=\frac{f_{is1,E}^{n}S_{\phi D}T_{p}^{2}}{2}$$

(29) $$Q_{33}=-\frac{S_{vN}T_{p}^{3}}{3R^{2}}+S_{\phi E}T_{p}+\frac{{\left({\Delta \Theta }_{is1,N}^{n}\right)}^{2}S_{\phi D}T_{p}}{3}$$

(30) $$Q_{34}=-\frac{f_{is1,N}^{n}{\Delta \Theta }_{is1,N}^{n}S_{\phi D}T_{p}^{3}}{8}+\frac{f_{is1,E}^{n}f_{is1,N}^{n}S_{\phi D}T_{p}^{5}}{20R}$$

(31) $$Q_{35}=-\frac{f_{is1,N}^{n}{\Delta \Theta }_{is1,N}^{n}S_{\phi D}T_{p}^{2}}{3}+\frac{f_{is1,E}^{n}f_{is1,N}^{n}S_{\phi D}T_{p}^{4}}{8R}$$

(32) $$Q_{36}=-\frac{{\Delta \Theta }_{is1,E}^{n}{\Delta \Theta }_{is1,N}^{n}S_{\phi D}T_{p}}{3}+\frac{\left(f_{is1,E}^{n}{\Delta \Theta }_{is1,E}^{n}-f_{is1,N}^{n}{\Delta \Theta }_{is1,N}^{n}\right)S_{\phi D}T_{p}^{3}}{8R}+\frac{f_{is1,E}^{n}f_{is1,N}^{n}S_{\phi D}T_{p}^{5}}{20R^{2}}$$

(33) $$Q_{39}=\frac{{\Delta \Theta }_{is1,N}^{n}S_{\phi D}T_{p}}{2}-\frac{f_{is1,E}^{n}S_{\phi D}T_{p}^{3}}{6R}$$

(34) $$Q_{44}=\frac{S_{vE}T_{p}^{3}}{3}-\frac{{\left(f_{is1,D}^{n}\right)}^{2}S_{\phi N}T_{p}^{5}}{20}+\frac{{\left(f_{is1,N}^{n}\right)}^{2}S_{\phi D}T_{p}^{5}}{20}$$

(35) $$Q_{45}=\frac{S_{vE}T_{p}^{2}}{2}-\frac{{\left(f_{is1,D}^{n}\right)}^{2}S_{\phi N}T_{p}^{4}}{8}+\frac{{\left(f_{is1,N}^{n}\right)}^{2}S_{\phi D}T_{p}^{4}}{8}$$

(36) $$Q_{46}=\frac{S_{vE}T_{p}^{3}}{3R}-\frac{f_{is1,D}^{n}S_{\phi N}T_{p}^{3}}{6}+\frac{f_{is1,N}^{n}{\Delta \Theta }_{is1,E}^{n}S_{\phi D}T_{p}^{3}}{8}$$

(37) $$Q_{49}=-\frac{f_{is1,N}^{n}S_{\phi D}T_{p}^{3}}{6}$$

(38) $$Q_{55}=S_{vE}T_{p}-\frac{{\left(f_{is1,D}^{n}\right)}^{2}S_{\phi N}T_{p}^{3}}{3}+\frac{{\left(f_{is1,N}^{n}\right)}^{2}S_{\phi D}T_{p}^{3}}{3}$$

(39) $$Q_{56}=\frac{S_{vE}T_{p}^{2}}{2R}-\frac{f_{is1,D}^{n}S_{\phi N}T_{p}^{2}}{2}+\frac{f_{is1,N}^{n}{\Delta \Theta }_{is1,E}^{n}S_{\phi D}T_{p}^{2}}{3}$$

(40) $$Q_{59}=-\frac{f_{is1,N}^{n}S_{\phi D}T_{p}^{2}}{2}$$

(41) $$Q_{66}=\frac{S_{vE}T_{p}^{3}}{3R^{2}}-S_{\phi N}T_{p}+\frac{{\left({\Delta \Theta }_{is1,E}^{n}\right)}^{2}S_{\phi D}T_{p}}{3}$$

(42) $$Q_{69}=-\frac{\Delta \Theta_{is1,E}^{n}S_{\phi D}T_{p}}{2}-\frac{f_{is1,N}^{n}S_{\phi D}T_{p}^{3}}{6R}$$

where $S_{vN},S_{vE},S_{vD}$ are power spectral densities (PSD) of the accelerometers noises, and $S_{\phi N},S_{\phi E},S_{\phi D}$ are power spectral densities of the gyroscopes noises in the *n-frame*. Initially, it was assumed that the power spectral densities of noises of the accelerometers and the gyroscopes were equal for all INS axes, and respective values were derived from the sensor manual [18]:

(43) $$S_{vN}=S_{vE}=S_{vD}\approx 1.218\cdot 10^{-11}\frac{rad^{2}}{s}\;S_{\phi N}=S_{\phi E}=S_{\phi D}\approx 1.365\cdot 10^{-6}\frac{m^{2}}{s^{3}}$$

In the integrated navigation system, the estimation of the reference system errors (INS) is possible due to the GPS receiver, which provides a redundancy of the measurement data. The process of integration, however, requires bringing INS and GPS measurements to a common moment of time and a point in space, which is neglected in solutions presented in the MOCO literature [1,2,3] but is considered in the method presented in this paper. The data synchronization was achieved by interpolating results obtained at a higher frequency [from the inertial measurement unit (IMU)] to the time of data obtained at a lower frequency (GPS data). In this method, an additional (*artificial*) IMU measurement was generated at a GPS data time ($t_{k,GPS}$), and then it was placed between two standard IMU measurements (taken at $t_{k-1,IMU}$ and $t_{k,IMU}$). Considering the high IMU update rate (1 kHz) and the low dynamics of the UAV, the specific force vector was interpolated using a zero-order polynomial, as it was assumed that the specific forces remained constant during a single measurement period:

(44) $$f_{is1}^{s1}\left(t_{k,GPS}\right)=f_{is1}^{s1}\left(t_{k,IMU}\right)$$

On the other hand, the delta angle vector $\Delta \Theta_{is1}^{s1}$, measured in time interval $\left(t_{k-1,IMU};t_{k,IMU}\right)$ and released at the moment $t_{k,IMU}$ is divided into two parts, $\Delta \Theta_{1}$ and $\Delta \Theta_{2}$, representing angular changes in the subintervals $\left(t_{k-1,IMU};t_{k,GPS}\right)$ and $\left(t_{k,GPS};t_{k,IMU}\right)$:

(45) $$\Delta \Theta_{is1}^{s1}\left(t_{k,IMU}\right)=\Delta \Theta_{1}+\Delta \Theta_{2}$$

(46) $$\Delta \Theta_{1}=\Delta \Theta_{is1}^{s1}\left(t_{k,IMU}\right)\cdot \frac{t_{k,GPS}-t_{k-1,IMU}}{t_{k,IMU}-t_{k-1,IMU}}$$

(47) $$\Delta \Theta_{2}=\Delta \Theta_{is1}^{s1}\left(t_{k,IMU}\right)\cdot \frac{t_{k,IMU}-t_{k,GPS}}{t_{k,IMU}-t_{k-1,IMU}}$$

**Figure 1:**
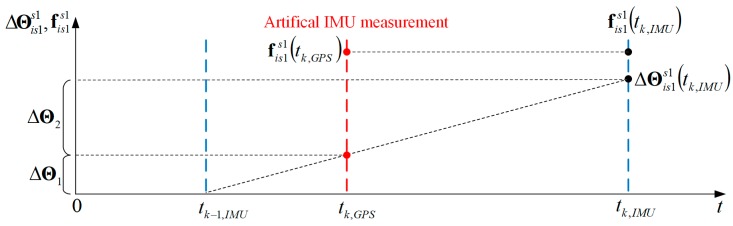
Inertial Measurement System (INS) data interpolation.

The interpolation procedure described above is shown in Figure 1.

In the next step, the INS result at $t_{k,GPS}$ time was calculated using the interpolated IMU data, i.e., $f_{is1}^{s1}\left(t_{k,GPS}\right)$ and $\Delta \Theta_{1}$. At the moment of GPS data time, the Kalman filter procedure presented in the following section was also executed. The INS solutions at the time $t_{k,IMU}$ were calculated using INS results from the *artificial* time $t_{k,GPS}$, the measured specific force vector $f_{is1}^{s1}\left(t_{k,IMU}\right)$ from the time $t_{k,IMU}$, and the remaining delta angle vector $\Delta \Theta_{2}$.

Due to different mounting points of the GPS antennas and the IMU onboard the UAV, the measured data initially referred to different points in space. A translation of the results to a common point—a body frame (*b-frame*)—origin was realized by converting the measured GPS antenna position $r_{A1}^{e,GPS}$ expressed in the Earth-Centered Earth-Fixed reference frame (*e-frame*) to the *b-frame* origin position $r_{b}^{e,GPS}$ using known lever arm $l_{b,A1}^{b}$ between the *b-frame* origin and this antenna:

(48) $$r_{b}^{e,GPS}=r_{A1}^{e,GPS}-C_{n}^{e}C_{b}^{n}l_{b,A1}^{b}$$

where $C_{b}^{n}$ converts a vector from the *b-frame* to the *n-frame*, and $C_{n}^{e}$ converts a vector from the *n-frame* to the *e-frame*.

In the MOCO using an integrated INS/GPS system, there is a common assumption that INS and GPS produce data at the same frequency, which simplifies the construction of the measurement vector z(k) [1,2,3]. In our system, the GPS position was determined at 5 Hz, while GPS velocity was at 20 Hz. To use these measurements with their maximal frequencies, the measurement vector was divided into two vectors, a vector of position difference $z_{r}\left(k\right)$ and a vector of velocity difference $z_{v}\left(k\right)$:

(49) $$z\left(k\right)=z_{r}\left(k\right)+z_{v}\left(k\right)=\left[\begin{array}{c} \varphi_{b}^{INS}-\varphi_{b}^{GPS}\\ 0\\ \lambda_{b}^{INS}-\lambda_{b}^{GPS}\\ 0\\ h_{b}^{INS}-h_{b}^{GPS}\\ 0 \end{array}\right]+\left[\begin{array}{c} 0\\ v_{is1,N}^{n,INS}-v_{A1,N}^{n,GPS}\\ 0\\ v_{is1,E}^{n,INS}-v_{A1,E}^{n,GPS}\\ 0\\ v_{is1,D}^{n,INS}-v_{A1,D}^{n,GPS} \end{array}\right]$$

where $\varphi_{b}$ is the latitude of the *b-frame* origin, $\lambda_{b}$ is its longitude, and $h_{b}$ is its altitude above the reference ellipsoid (the upper index describes the system used to determine the respective coordinate, INS or GPS), $\left[\begin{array}{ccc} v_{is1,N}^{n,INS} & v_{is1,E}^{n,INS} & v_{is1,D}^{n,INS} \end{array}\right]$ is the velocity vector of the *s-frame* with reference to the *i-frame* expressed in the *n-frame* and measured by the INS, whereas $\left[\begin{array}{ccc} v_{A1,N}^{n,GPS} & v_{A1,E}^{n,GPS} & v_{A1,D}^{n,GPS} \end{array}\right]$ is an analogous velocity vector measured by the GPS receiver. This solution allowed us to use maximal sensor output update rates because—due to the independent treatment of velocity and position measurements—there was no need to limit the rate of velocity data to the rate of position data. The proposed approach, however, makes it impossible to use a classical Kalman filter algorithm, and therefore a sequential Kalman filter was used.

In the considered INS/GPS system, the observation matrix H(k) is given as follows:

(50) $$H\left(k\right)=\left[\begin{array}{ccccccccc} \frac{1}{R+h_{b}^{INS}} & 0 & 0 & 0 & 0 & 0 & 0 & 0 & 0\\ 0 & 1 & 0 & 0 & 0 & 0 & 0 & 0 & 0\\ 0 & 0 & 0 & \frac{1}{\left(R+h_{b}^{INS}\right)\cos\left(\varphi_{b}^{INS}\right)} & 0 & 0 & 0 & 0 & 0\\ 0 & 0 & 0 & 0 & 1 & 0 & 0 & 0 & 0\\ 0 & 0 & 0 & 0 & 0 & 0 & -1 & 0 & 0\\ 0 & 0 & 0 & 0 & 0 & 0 & 0 & 1 & 0 \end{array}\right]$$

and the measurement errors covariance matrix R can be described using the formula:

(51) $$R=\mathrm{diag}\left(\left[\begin{array}{cccccc} \sigma_{\varphi }^{2} & \sigma_{vN}^{2} & \sigma_{\lambda }^{2} & \sigma_{vE}^{2} & \sigma_{h}^{2} & \sigma_{vD}^{2} \end{array}\right]\right)$$

where $\sigma_{\varphi }^{2}$, $\sigma_{\lambda }^{2}$, $\sigma_{h}^{2}$ are the variances of latitude, longitude, and altitude errors, and $\sigma_{vN}^{2}$, $\sigma_{vE}^{2}$, $\sigma_{vD}^{2}$ are the variances of north, east, and down velocity errors of the GPS data. These variances were determined in our system during a 1 min pre-launch measurement session. The determined values of position and velocity errors standard deviations used to create the **R** matrix were as follows: $\sigma_{\varphi }=5.0364\cdot 10^{-10}rad$, $\sigma_{\lambda }=2.6561\cdot 10^{-10}rad$, $\sigma_{h}=4.0899\cdot 10^{-3}m$, $\sigma_{vN}=2.0115\cdot 10^{-2}\frac{m}{s}$, $\sigma_{vE}=1.1904\cdot 10^{-2}\frac{m}{s}$, $\sigma_{vD}=2.9508\cdot 10^{-2}\frac{m}{s}$.

## 3. Kalman Filter

**Figure 2:**
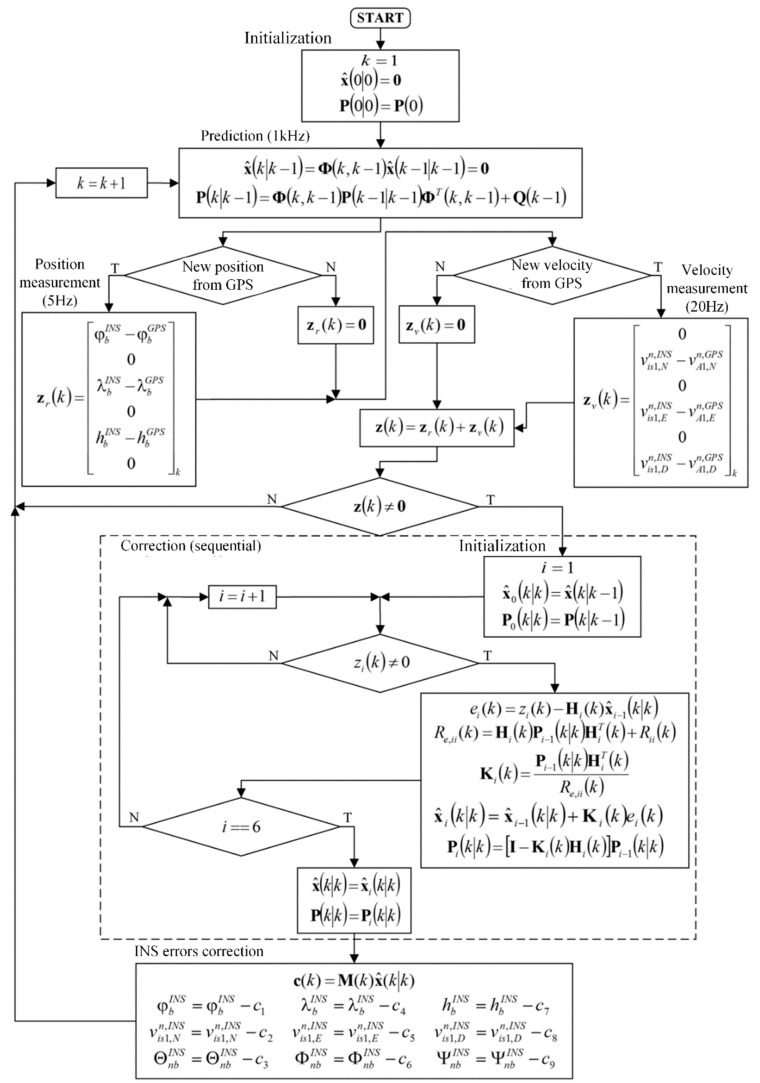
Sequential Kalman filter for INS/Global Positioning System (GPS) system with feed-backward correction.

An algorithm of sequential Kalman filter used in the proposed system consisted of four stages: initialization, state prediction, measurement update, and reference system (INS) correction (Figure 2). In the initialization phase, the initial values of the system state vector elements were assumed to be zeroes, since the INS was initialized using the GPS data with zero-mean errors. Moreover, the initial value of the covariance matrix P(0|0) was determined using the formula [12,13]:

(52) $$P\left(0|0\right)=P\left(0\right)=\;\mathrm{diag}\left(\left[\begin{array}{ccc} \sigma_{\varphi }^{2}{\left(R+h_{b}^{INS}\right)}^{2} & \sigma_{vN}^{2} & \begin{array}{ccc} \sigma_{\phi E}^{2} & \sigma_{\lambda }^{2}{\left(\left(R+h_{b}^{INS}\right)\cos\left(\varphi_{b}^{INS}\right)\right)}^{2} & \begin{array}{ccc} \sigma_{vE}^{2} & \sigma_{\phi N}^{2} & \begin{array}{ccc} \sigma_{h}^{2} & \sigma_{vD}^{2} & \sigma_{\phi D}^{2} \end{array} \end{array} \end{array} \end{array}\right]\right)$$

where the elements $P_{11},P_{44},P_{77}$ are the GPS position errors variances expressed in $m^{2}$ units converted from $rad^{2}$, $P_{22},P_{55},P_{88}$ are the GPS velocity errors variances, and $P_{33},P_{66},P_{99}$ represent the initial variances of INS orientation errors, which could be determined using the IMU documentation or a dedicated experiment.

The state prediction phase was executed at the INS update rate (1 kHz). In the presented filter working in the INS/GPS system with a feed-backward correction, the errors of the reference system were reset after each measurement update; therefore, in the prediction phase, the a priori state vector $\hat{x}\left(k|k-1\right)$ elements had zero values [11].

The measurement update (correction) phase was triggered by either the GPS position or the GPS velocity data. In the case of acquiring only GPS position information (which occurred with the frequency of 5 Hz), the measurement vector had non-zero elements in the positions i∈{1,3,5}. In the case of acquiring GPS velocity data, the z vector had non-zero elements in the positions i∈{2,4,6}, whereas after acquiring both position and velocity data, all the elements of this vector were non-zeroes.

In the sequential Kalman filter, the measurement update equations are represented in a scalar form, contrary to a matrix form in the classic Kalman filter. As a result, the calculations were performed independently and successively for each acquired element of the measurement vector. The measurement update began with an initialization using prediction results: ${\hat{x}}_{0}\left(k|k\right)=\hat{x}\left(k|k-1\right)$, $P_{0}\left(k|k\right)=P\left(k|k-1\right)$. Then, elements of the residual vector e, diagonal elements of the innovation covariance matrix $R_{e}$, and columns of Kalman gains matrix K were determined in a loop [11]. Based on them, the estimate of the state vector ${\hat{x}}_{i}\left(k|k\right)$ and the covariance matrix $P_{i}\left(k|k\right)$ were calculated after each *i-th* inner loop iteration [9]. After processing all the non-zero elements of the measurement vector, the correction phase ended, and the results obtained in the inner loop were assigned to the final measurement update results: $x\left(k|k\right)=x_{6}\left(k|k\right)$ and $P\left(k|k\right)=P_{6}\left(k|k\right)$.

The last phase of the navigation data processing was the INS errors correction using a feedback loop. The vector of corrections c(k) was calculated using the following equation:

(53) $$c\left(k\right)=M\left(k\right)\hat{x}\left(k|k\right)$$

where M is a matrix, which ensures appropriate translation from the *n-frame* to the *e-frame*:

(54) $$M\left(k\right)=\mathrm{diag}\left(\left[\begin{array}{ccccccccc} \frac{1}{R+h_{b}^{INS}} & 1 & 1 & \frac{1}{\left(R+h_{b}^{INS}\right)\cos\left(\varphi_{b}^{INS}\right)} & 1 & 1 & -1 & 1 & 1 \end{array}\right]\right)$$

The vector of corrections c(k) was subtracted from the current values of the navigation elements determined by the INS, and therefore the system errors were bounded, and the Kalman filter estimated only the residual errors (i.e., what remained after the previous correction).

## 4. Filter Tuning

The presented model of INS/GPS navigation system is based on an idealized and simplified description of errors occurring in the real system. Both the simplifications and the incomplete knowledge of the system make the developed Kalman filter algorithm not optimal. In such cases, the filter tuning procedure is commonly used, which involves modifying the model parameters in order to minimize the state vector estimation errors.

During this procedure, the values of Q and R matrices were modified in order to obtain the conformity of the statistical parameters of the residual vector e, which was a realization of a stochastic process of innovations and the theoretical parameters in the innovations process [19]. In the optimal Kalman filter, the innovations vector has a normal distribution with a zero mean [19]. The diagonal elements of the $R_{e}$ matrix represent the variances of the process components. According to the normal distribution properties, 95.5% of samples for each component of the residual vector e should lie within a range defined by ±2 standard deviations of the innovations process $\left(-2\sqrt{R_{e,ii}};2\sqrt{R_{e,ii}}\right)$.

In case of the Q matrix, the tuning procedure was applied to the power spectral densities of the IMU accelerometers and gyroscopes noises given in Equation (43). Common scaling coefficients $S_{coef,N}$, $S_{coef,E}$, $S_{coef,D}$ were used for accelerometers and gyroscopes for each axis. In the case of the R matrix, the scaled parameters were the variances of the GPS position and the velocity errors given in Equation (51). One common coefficient $R_{coef,pos}$ was used for all the position errors, and one coefficient $R_{coef,vel}$ was used for the velocity errors. Firstly, and $R_{coef,vel}$ were determined, as they were connected with the GPS system performance, which could be assumed to be repeatable in similar testing conditions. Then, further tuning was performed using Q matrix coefficients. The values of the coefficients established in the tuning procedure are presented in Table 1.

**Figure 3:**
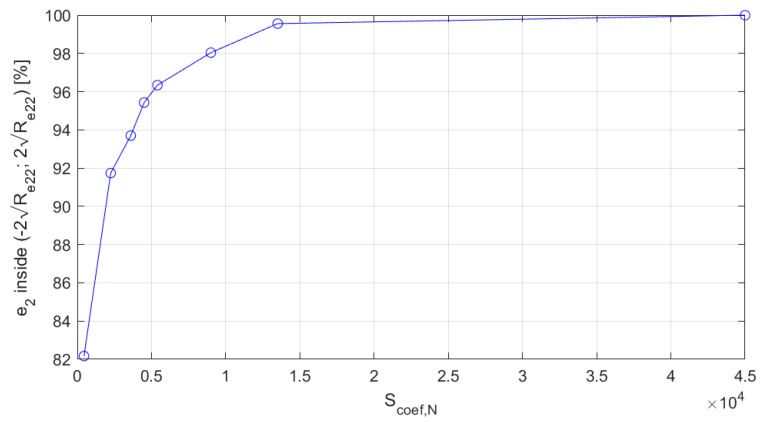
Influence of the $S_{coef,N}$ coefficient on the e_2_ statistics.

Table 2 shows the influence of the scaling coefficients on the e vector statistical parameters. The highlighted rows represent the results obtained with the use of the coefficients from Table 1. In this experiment, the influence of each coefficient was tested separately. Moreover, to give a better insight into the effects caused by changes of these coefficients in a wider ranges, Table 2 contains a more detailed analysis of the system sensitivity to the $S_{coef,N}$, which is also presented in Figure 3 for a chosen element of the e vector, namely, $e_{2}$. The results presented in this figure show that the number of $e_{2}$ samples lying within the $\left(-2\sqrt{R_{e,ii}};2\sqrt{R_{e,ii}}\right)$ range increased monotonically with the increase of the $S_{coef,N}$ coefficient, asymptotically tending to 100%. Similar behavior was also observed for other coefficients and the residual vector elements.

Choosing appropriate values of the scaling coefficients is an experimental process, and sometimes achieving the target of 95.5% samples within the ± 2 standard deviations range for each component of the **e** vector is impossible due to the limited number of scaling coefficients. However, the proposed set of coefficients was enough to ensure that all the residual vector values approximately had the desired statistical parameters, which is visible in Table 1. A representative analysis of the $e_{2}$ component sensitivity to the *S_coef,N_* showed that ±20% deviations of the $S_{coef,N}$ from its initially set value (Table 1) had minor influence on the $e_{2}$ statistics (the total count of samples outside the ± 2 standard deviations range changed from 93.7% to 96.3%, which was close to the desired value of 95.5%). This relatively small sensitivity is useful in real-world conditions, because changes of the IMU parameters (over time or between different units) should not have a significantly adverse effect on the system’s performance.

**Figure 4:**
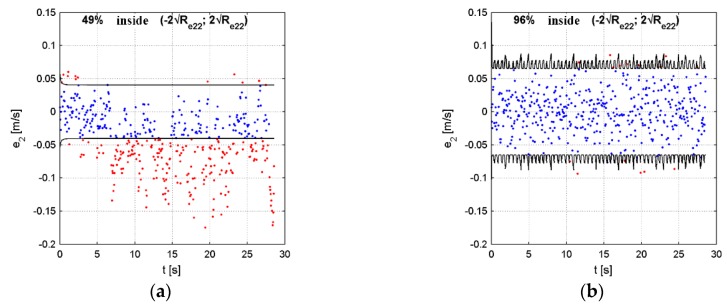
The $e_{2}$ element of the residual vector: (**a**) before tuning, (**b**) after tuning.

As shown in Figure 4, where $e_{2}$ component is once again presented as an example, initially, the statistical condition was not met; however, after the tuning procedure, the desired statistical parameters were obtained.

## 5. Results of INS/GPS System Testing

**Figure 5:**
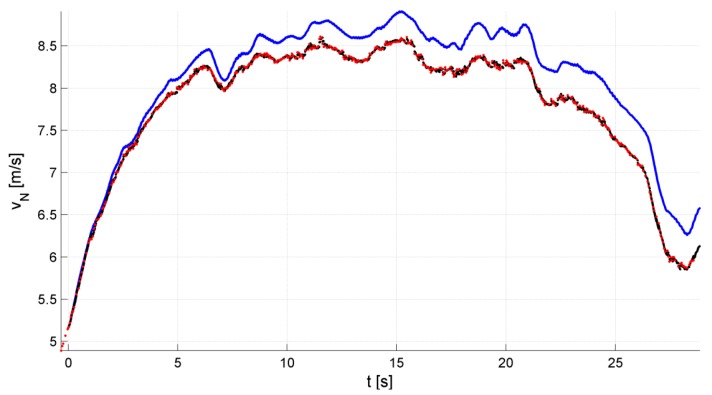
Velocity along north axis: black—INS/GPS, red—GPS, blue—INS.

**Figure 6:**
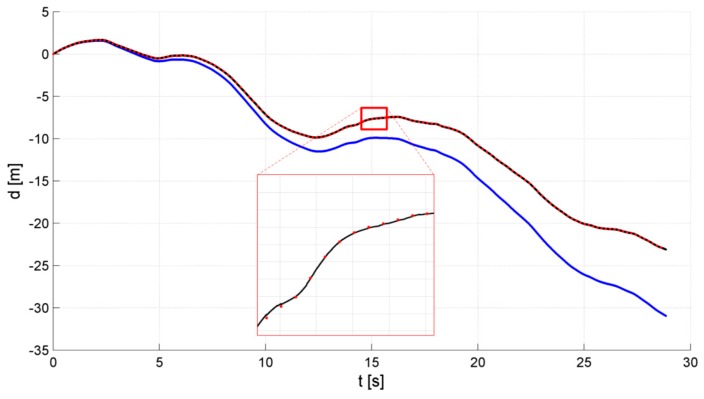
Position along vertical (down) axis: black—INS/GPS, red—GPS, blue—INS.

During a test campaign, the whole SAR system—which was named WATSAR [17,20] and was designed by the authors, among others—was tested in flight for various UAV trajectories and for various system configurations. Detailed results of those tests were presented in [17,20,21,22,23]. Apart from the online data processing, which was realized in our system, both radar and navigation data were registered for the post-mission offline processing. Chosen results of this offline processing of IMU and GPS data with the use of our Kalman filter are presented in Figure 5 and Figure 6. The results of calculation or estimation of one velocity component (along the north axis) and one position component (along the vertical axis) were selected as representative for the results obtained for all axes.

The obtained results indicate that the integration of INS and GPS data allowed us to eliminate the effect of the INS system error growth, making the integrated system a potentially good source of navigational corrections for the MOCO procedure. In addition, the INS/GPS system update rate was greater than the rate obtained using the GPS alone. The output from the integrated navigation system was then loaded into the navigation correction algorithm, as presented in [21].

## 6. Results of MOCO Testing

**Figure 7:**
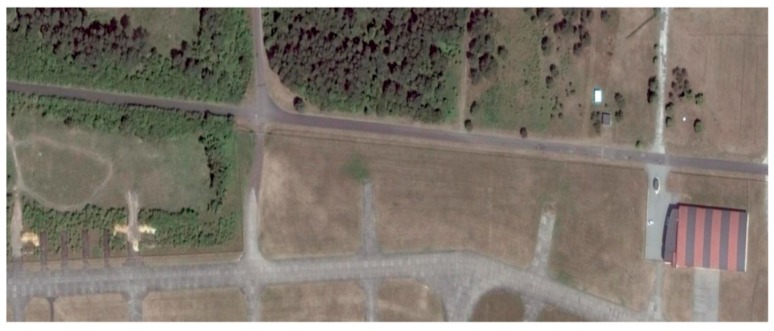
Aerial photography of the imaged area.

**Figure 8:**
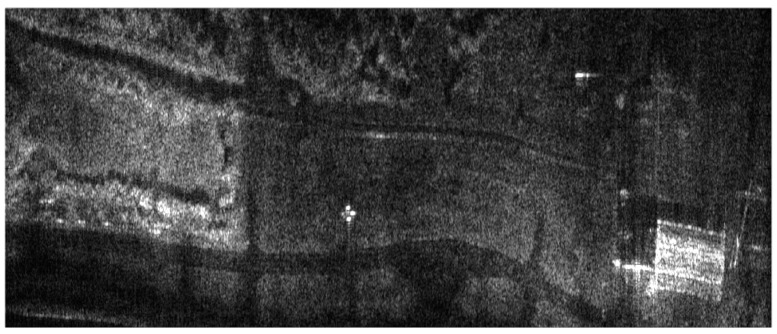
Radar terrain image obtained without navigation correction.

**Figure 9:**
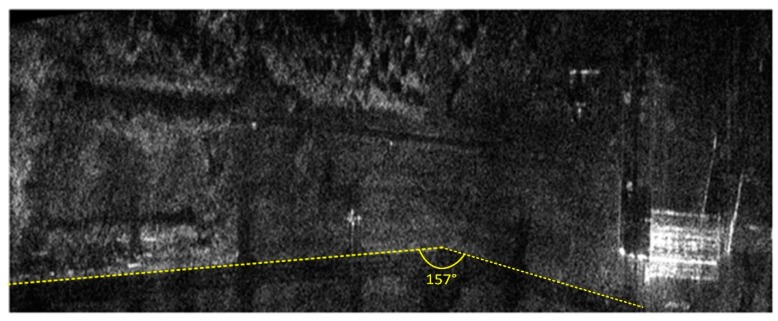
Radar terrain image obtained with the use of INS data; yellow line—a north edge of the taxiway determined using INS-based image.

**Figure 10:**
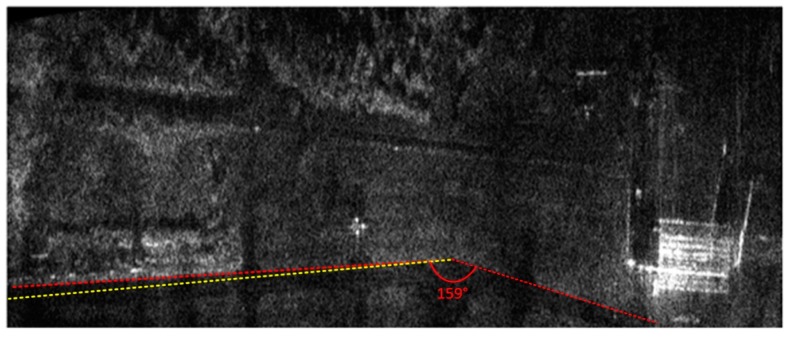
Radar terrain image obtained with the use of INS/GPS data; yellow line—a north edge of the taxiway determined using INS-based image, red line—a north edge of the taxiway determined using INS/GPS-based image.

The influence of the navigation corrections obtained from the proposed INS/GPS system on SAR terrain images was determined using the WATSAR aerial system equipped with a *Ku* band (15.9 GHz) synthetic aperture radar [20]. The imaged area is shown in Figure 7, an image obtained without the use of navigation correction is presented in Figure 8, the image shown in Figure 9 was obtained using corrections originating from the INS system, while Figure 10 contains an image calculated using the INS/GPS system data. During the imaging, an aperture length of 501 elements and a rectangular window were used.

Both the INS/GPS and the INS corrections enabled a significant reduction of the image geometric distortions in relation to the image obtained without MOCO. Between these images, however, there were significant differences in the efficiency of this reduction, which is clearly visible in the southwest part of the airfield taxiway (left, bottom part of Figure 7, Figure 8, Figure 9 and Figure 10). In Figure 9 (with INS corrections), the north edge of the taxiway is indicated by a yellow dotted line. This edge deviates in the southern direction in relation to the analogous edge determined in the INS/GPS image (Figure 10), which is marked as a red line. For comparison purposes, the yellow line from Figure 9 was translated the Figure 10. In the INS corrected image, the erroneous deviation of the taxiway is visible in the final part of the measurement session (the flight was carried from the east to the west) and was related to a growing discrepancy between the INS and the real trajectory due to increasing INS errors. To evaluate the system’s ability to reduce the geometric distortions, the angle between the two parts of the taxiway was compared to the true one, which equals 159°. The reference angle was measured using an aerial image and image processing software, where three points located on the north edge of the taxiway were determined in order to produce two intersected lines. In the case of the INS-based image, this angle equaled 157°, whereas in the image obtained using INS/GPS system, the angle matched the true one and was 159°. This leads to the conclusion that the INS-based MOCO can be used only for time-limited measurement sessions. On the contrary, the INS/GPS MOCO procedure provides a better reduction of geometric distortions and can be used without these time limitations, thus it could be an attractive solution for SAR imaging MOCO.

It should be noted that geometric distortions of images can be even better assessed using Ground Control Points (GCPs), which must be visible in the radar terrain image and in the reference image (often prepared in the visual spectrum range) [24,25,26]. In this method, the distortions can be expressed as a standard deviation of the radar image GCPs coordinates in relation to the reference image. The obvious limitation of this method is the need for a reference image and the set of GCPs with precisely determined coordinates. In the experiments presented in this paper, there were no GCPs, thus usage of this method was not possible. However, in the future tests, the GCPs are planned to be deployed in the test area to improve the system’s evaluation.

Besides geometric distortions, in our research, we assessed the influence of various MOCO procedures on the quality of SAR images using techniques typically used in image quality assessment [4,5,8,27,28]. Contrast, entropy, and various parameters describing image spatial resolution were calculated for the images presented in Figure 8, Figure 9 and Figure 10.

Due to the use of INS/GPS based MOCO, the test image had a better (higher) contrast (IC) than the image obtained without the correction (IC = 7.56 compared to 4.06). On the other hand, this result was worse (lower) than the image contrast obtained with the use of INS-based MOCO (IC = 8.32) [5,8]. A similar effect related to the image entropy (E). The lower the entropy was, the better the image was focused [8]. Due to the use of INS/GPS corrections, the entropy of the test image dropped from 14.43 (the image without MOCO) to 13.94. Again, this result was slightly worse than the entropy of the image obtained with the use of INS-based MOCO (E=13.83).

**Figure 11:**
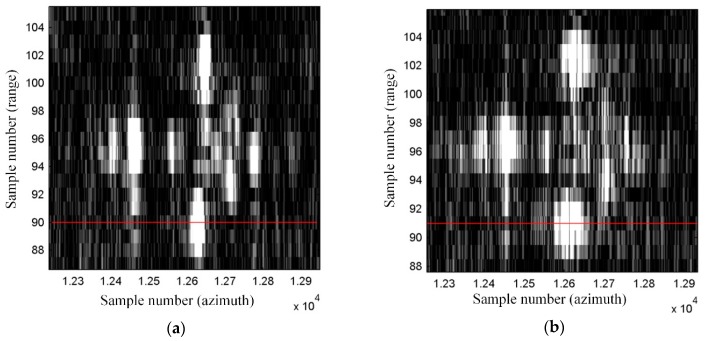
Image of the arrow made of corner reflectors: (**a**) with the INS-based motion compensation (MOCO), (**b**) with the INS/GPS-based MOCO; red line—an analyzed row.

To determine the spatial resolution (SR) [4], the peak sidelobe ratio (PSLR), and the integrated sidelobe ratio (ISLR) [27,28], a horizontal line from a bottom part of the image was chosen. This line passed through one of the corner reflectors, which were formed in an arrow-shape and were placed on the apron during testing of our SAR system. An enlarged image of that arrow is shown in Figure 11. The image obtained with the use of the INS/GPS-based corrections (right side) was more blurred, and the objects forming the arrow were harder to distinguish compared to the INS-based MOCO image (left side).

**Figure 12:**
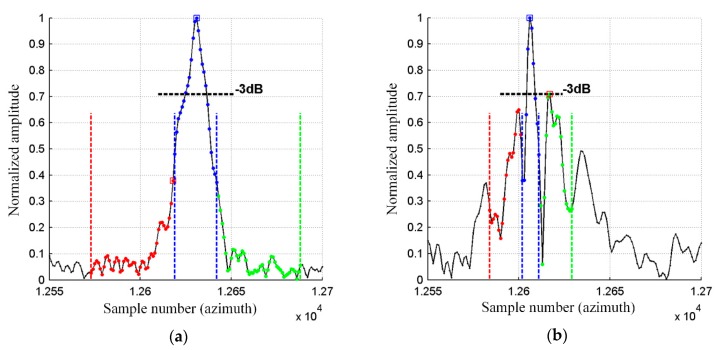
Main lobe (blue line) and side lobes (red and green lines) of the selected corner reflector synthetic aperture radar (SAR) image: INS-based MOCO (**a**), INS/GPS based MOCO (**b**).

**Figure 13:**
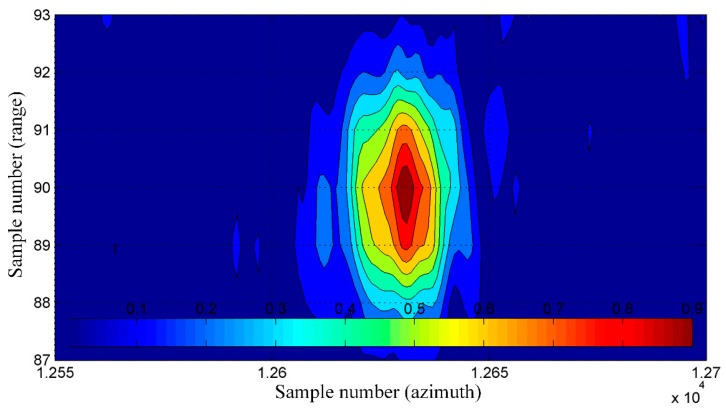
The normalized amplitude of the selected corner reflector pixels—the INS-based MOCO.

**Figure 14:**
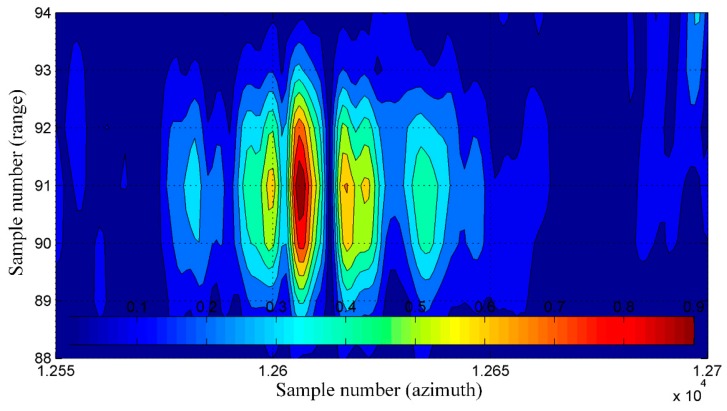
The normalized amplitude of the selected corner reflector pixels—the INS/GPS-based MOCO.

A graph of a normalized amplitude of image pixels lying on the mentioned line is shown in Figure 12. For an even more intuitive visualization, normalized amplitudes of image pixels lying on several lines containing the considered corner reflector are presented in Figure 13 and Figure 14 as colored contour plots.

In the case of the INS/GPS-based MOCO image (Figure 12b and Figure 14), the main lobe was narrower than in the image obtained using only the INS (Figure 12a and Figure 13), which in theory leads to a better spatial resolution (SR = 0.110 m versus SR = 0.304 m). In practice, however, in the INS/GPS image, the main lobe was divided, which led to side lobes with very high amplitudes. These side lobes may have been interpreted as false objects, which would have worsened the resolution, causing the image to be blurred.

In the MOCO using INS/GPS data, the high-level side lobes led to a significant deterioration (which meant the increase of the value) of the PSRL and the ISLR compared not only to the image obtained with the INS corrections (PSLR = −8.45 dB, ISLR = −10.5 dB) but also to the image obtained without the MOCO procedure (PSLR = −5.17 dB, ISLR = −5.85 dB). In the case of the INS/GPS method, the obtained results were: PSLR = −2.99 dB, ISLR = 1.52 dB. A comparison of these and the previously mentioned results is given in Table 3. The arrows in the brackets following the symbols of respective parameters show the desirable direction of changes of these parameters resulting in a better image quality.

**Figure 15:**
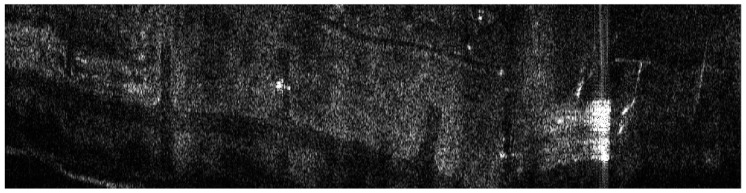
Radar terrain image obtained without MOCO.

**Figure 16:**
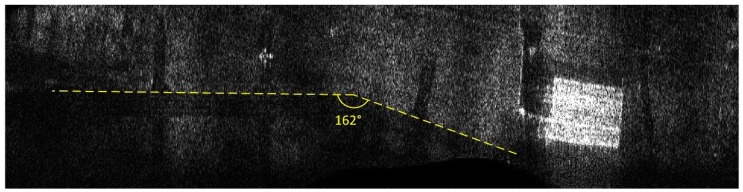
Radar terrain image obtained with the use of INS data; yellow line—a north edge of the taxiway.

**Figure 17:**
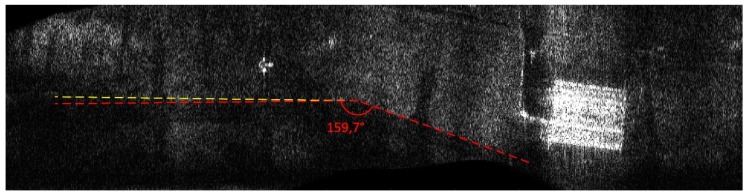
Radar terrain image obtained with the use of INS/GPS data; yellow line—a north edge of the taxiway determined using INS-based image, red line—a north edge of the taxiway determined using INS/GPS-based image.

Similar experiments and analyses were conducted for various terrain scans. Results obtained for another flight are presented in Figure 15 (without MOCO), Figure 16 (INS-based MOCO), and Figure 17 (INS/GPS-based MOCO), while Table 4 contains an assessment of the quality parameters.

In the case of the image obtained without MOCO (Figure 15), it was not possible to determine the SR, the PSLR, and the ISLR parameters due to significant image distortions, which hindered distinguishing individual corner reflector. As in the previous example, however, the INS/GPS-based MOCO was more effective in the reduction of the geometric distortions than the INS-based method. In the INS/GPS image, the shape of the taxiway is closer to the true one due to the fact that the north edge of the taxiway in the INS image deviates to the north. Moreover, the shape of the hangar visible on the right (east) side of the INS/GPS image is rectangular, while in the INS image, it is distorted and becomes trapezoidal with a visible nonlinearity of its southern edge.

Considering the superiority of the INS/GPS-based approach in terms of its ability to reduce geometric distortions, it was necessary to investigate the reasons that led to the generally worse performance in terms of the quality parameters presented in Table 3 and Table 4.

**Figure 18:**
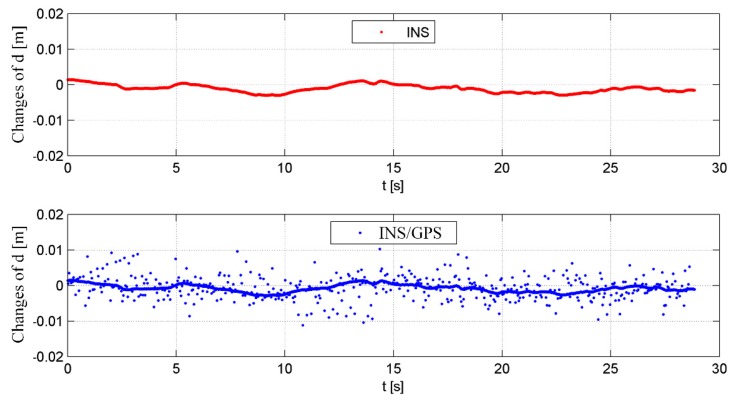
A comparison of changes of the vertical component of the unmanned aerial vehicles (UAV) position vector in one measurement session for the INS and the INS/GPS systems.

The reasons for these phenomena were random errors associated with the GPS receiver. In the INS, the UAV position and the velocity changes were small in one measurement period (1 ms), while in the GPS-only data, abrupt changes occurred resulting from the GPS errors characteristics. These GPS random errors were subsequently transferred to the INS/GPS integrated solution. As an example, in Figure 18, the changes of the vertical position component calculated for INS and INS/GPS for one measurement session are presented.

In the case of the INS, the calculated position varied by a maximum of 2.9 mm between two consecutive samples. A similar trend was visible in the INS/GPS data (as a bold line). The points belonging to this line corresponded to the estimation results in which there was no INS error correction by a Kalman filter. The measurement update realized in the Kalman filter with a lower frequency than the calculation of INS solutions led to rapid changes of the estimated navigation parameters, which were visible as the points separated from the main trend line (Figure 15). These disturbances propagated through the navigation corrections and the MOCO procedure to the SAR image processor, which increased the echo signal sidelobes amplitude (Figure 12b and Figure 14). In the final image, this was visible as blurs and false targets [29,30].

## 7. Discussion

The article presents an integrated INS/GPS system with a dedicated Kalman filter algorithm, which considers a time difference between the IMU and the GPS updates and a frequency difference between the position and the velocity updates from the GPS receiver—problems usually neglected in available literature. The problem of time delay between the IMU and the GPS data was solved using an artificial IMU measurement determined for the GPS data time. A sequential Kalman filter algorithm was used to process the GPS position and the velocity data separately with their maximum available rates.

The results of the experiments conducted by the authors show that the main advantage of the proposed INS/GPS approach is its ability to reduce image geometric distortions, which is more effective than in competitive solutions. This allows the user to conduct long-term observation sessions, because there is no time-limitation due to the INS error growth, which can be crucial in real-world scenarios. In such a system, the INS/GPS method can be used for current image analysis, whereas the INS-based approach can be triggered only occasionally and for short periods to evaluate small parts of the images in more detail. The analyses presented in the paper may be helpful for researchers and engineers in deciding when and how to apply an INS/GPS-based MOCO method in a SAR system and in properly assessing its influence on the quality of the obtained radar images.

## Figures and Tables

**Table 1:** **Q** and **R** matrices scaling coefficients.

**Scaling Coefficient**	$R_{coef,pos}$	$R_{coef,vel}$	$S_{coef,N}$	$S_{coef,E}$	$S_{coef,D}$
**Value**	10.25	1.0	$4.5\cdot 10^{3}$	$1.1\cdot 10^{3}$	$2.3\cdot 10^{4}$

**Table 2:** Influence of the tuned parameters on the **e** vector statistical properties.

Coefficient Value	$\mathrm{Percentage}\;\mathrm{of}\;\mathrm{the}\;e\;\mathrm{Vector}\;\mathrm{Components}\;\mathrm{Samples}\;\mathrm{Which}\;\mathrm{Lie}\;\mathrm{Within}\;\left(-2\sqrt{R_{e,ii}};2\sqrt{R_{e,ii}}\right)$					
	$e_{1}$	$e_{2}$	$e_{3}$	$e_{4}$	$e_{5}$	$e_{6}$
$0.5\cdot R_{coef,pos}$	85	95	97	96	88	94
$1.0\cdot R_{coef,pos}$	92	96	98	95	93	95
$1.5\cdot R_{coef,pos}$	95	95	100	95	96	95
$0.5\cdot R_{coef,vel}$	88	90	95	86	87	92
$1.0\cdot R_{coef,vel}$	92	96	98	95	93	95
$1.5\cdot R_{coef,vel}$	96	97	99	98	96	97
$0.1\cdot S_{coef,N}$	91.67	82.17	98.41	96.09	92.56	95.00
$0.5\cdot S_{coef,N}$	90.28	91.74	98.41	95.47	92.56	95.00
$0.8\cdot S_{coef,N}$	90.97	93.70	98.41	95.47	92.56	95.00
$1.0\cdot S_{coef,N}$	91.67	95.63	98.41	95.47	92.56	95.00
$1.2\cdot S_{coef,N}$	91.67	96.30	98.41	96.09	92.56	95.00
$2.0\cdot S_{coef,N}$	91.67	98.04	98.41	96.09	92.56	95.00
$3.0\cdot S_{coef,N}$	94.67	99.56	98.41	96.09	92.56	95.00
$10.0\cdot S_{coef,N}$	97.22	100.00	98.41	96.09	92.56	95.00
$0.1\cdot S_{coef,E}$	91	94	97	89	93	95
$1.0\cdot S_{coef,E}$	92	96	98	95	93	95
$10\cdot S_{coef,E}$	91	95	99	100	93	95
$0.1\cdot S_{coef,D}$	91	95	98	95	90	83
$1.0\cdot S_{coef,D}$	92	96	98	95	93	95
$10\cdot S_{coef,D}$	92	95	98	95	100	100

**Table 3:** Parameters of the quality of the selected SAR image.

Source of Corrections	Parameter of Image Quality				
	IC (↑)	E (↓)	SR [m] (↓)	PSLR [dB] (↓)	ISLR [dB] (↓)
**none**	4.06	14.43	0.913	−5.17	−5.85
**INS**	8.32	13.83	0.304	−8.45	−10.50
**INS/GPS**	7.56	13.94	0.119	−2.99	1.52

IC—image contrast, E—entropy, SR—spatial resolution, PSLR—peak sidelobe ratio, ISLR—integrated sidelobe ratio.

**Table 4:** Parameters of the quality of the selected SAR image.

Source of Corrections	Parameter of Image Quality				
	IC (↑)	E (↓)	SR [m] (↓)	PSLR [dB] (↓)	ISLR [dB] (↓)
**none**	4.69	13.83	–	–	–
**INS**	6.00	13.01	0.195	−4.12	−2.58
**INS/GPS**	5.78	12.96	0.366	−1.92	−0.40
